# Sr_3_(Al_3+*x*_Si_13−*x*_)(N_21−*x*_O_2+*x*_):Eu^2+^ (*x* ∼ 0): a monoclinic modification of Sr-sialon

**DOI:** 10.1107/S1600536810003223

**Published:** 2010-01-30

**Authors:** Nobuo Ishizawa, Minami Kamoshita, Koichiro Fukuda, Kousuke Shioi, Naoto Hirosaki

**Affiliations:** a Nagoya Institute of Technology, Gokiso, Nagoya 466-8555, Japan; b National Institute for Materials Science, Namiki 1-1, Tsukuba, Ibaraki 305-0044, Japan

## Abstract

The structure of the title compound, Sr-bearing oxonitrido­aluminosilicate (Sr-sialon), contains two types of channels running along the *a* axis, with the three unique Sr atoms (coordinatioon number seven) residing in the larger one. The channels cross a three-dimensional Si–Al–O–N network, in which the Si and Al atoms are in a tetra­hedral coordination with N and O atoms. The chemical composition of the crystal is close to Sr_3_Al_3_Si_13_N_21_O_2_ (tris­trontium trialuminium trideca­silicon henicosa­nitride dioxide), which can be expressed as a mixture of SrSiN_2_, Si_3_N_4_, AlN, and SiO_2_ components in the molar ratio 3:3:3:1. The crystal studied was metrically orthorhombic, consisting of four twin components related by metric merohedry.

## Related literature

For the isotypic Eu-bearing oxonitridoaluminosilicate (Eu-sialon) Eu_3_Al_3+*x*_Si_13-*x*_N_21-*x*_O_2+*x*_ (*x *= 1/3), see: Michiue *et al.* (2009 ▶). For the closely related Sr-bearing sialon structure Sr_5_Al_5+*x*_Si_21-*x*_N_35-*x*_O_2+*x*_:Eu^2+^ (*x* ∼ 0), see: Oeckler *et al.* (2009 ▶). For the twin analysis, see: Coch (2004 ▶); Nespolo (2004 ▶). Ionic radii were taken from Shannon (1976 ▶).

## Experimental

### 

#### Crystal data

Sr_3_Al_3_Si_13_N_21_O_2_
*M* *_r_* = 2081.94
Monoclinic, 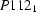

*a* = 14.7557 (5) Å
*b* = 7.4627 (2) Å
*c* = 9.0348 (3) Å
β = 90°
*V* = 994.89 (5) Å^3^
*Z* = 1
Mo *K*α radiation
μ = 9.05 mm^−1^
*T* = 296 K
0.08 × 0.08 × 0.01 mm

#### Data collection

Bruker APEXII CCD diffractometer
Absorption correction: numerical (de Meulenaer & Tompa, 1965 ▶) *T*_min_ = 0.532, *T*_max_ = 0.915
11854 measured reflections
4384 independent reflections
3672 reflections with *I* > 2σ(*I*)
*R*_int_ = 0.036

#### Refinement

*R*[*F* ^2^ > 2σ(*F* ^2^)] = 0.045
*wR*(*F* ^2^) = 0.105
*S* = 1.07
4384 reflections
162 parameters
1 restraint
Δρ_max_ = 1.33 e Å^−3^
Δρ_min_ = −1.25 e Å^−3^
Absolute structure: Flack (1983 ▶), 1942 Friedel pairs
Flack parameter: 0.00

### 

Data collection: *APEX2* (Bruker, 2007 ▶); cell refinement: *SAINT* (Bruker, 2007 ▶); data reduction: *SAINT*; program(s) used to solve structure: *SHELXS97* (Sheldrick, 2008 ▶); program(s) used to refine structure: *SHELXL97* (Sheldrick, 2008 ▶); molecular graphics: *ATOMS* (Dowty, 2008 ▶); software used to prepare material for publication: *publCIF* (Westrip, 2010 ▶).

## Supplementary Material

Crystal structure: contains datablocks I, global. DOI: 10.1107/S1600536810003223/br2134sup1.cif

Structure factors: contains datablocks I. DOI: 10.1107/S1600536810003223/br2134Isup2.hkl

Additional supplementary materials:  crystallographic information; 3D view; checkCIF report

## Figures and Tables

**Table 1:** Selected bond lengths (Å)

Sr1—N9	2.618 (13)
Sr1—N8	2.682 (13)
Sr1—N22	2.690 (16)
Sr1—N21	2.745 (14)
Sr1—N7	2.773 (9)
Sr1—N16	2.955 (10)
Sr1—N3	3.032 (10)
Sr2—N23^i^	2.557 (11)
Sr2—O17^ii^	2.573 (13)
Sr2—N21^iii^	2.702 (14)
Sr2—N4^i^	2.764 (14)
Sr2—N12^iv^	2.868 (7)
Sr2—N10	2.970 (8)
Sr2—N15	3.052 (10)
Sr3—O19^i^	2.549 (12)
Sr3—N22^v^	2.718 (16)
Sr3—N2	2.760 (7)
Sr3—N11^ii^	2.783 (13)
Sr3—N23^i^	2.851 (11)
Sr3—N6^iv^	2.994 (13)
Sr3—N1	3.096 (10)

Symmetry codes: (i) 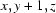; (ii) 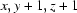; (iii) 
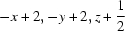
; (iv) 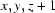; (v) 
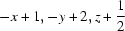
.

**Fig. 1.**
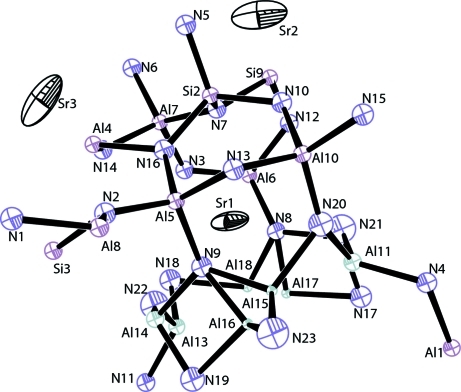
The asymmetric unit of the structure ; the displacement ellipsoids are plotted at the 80% probability level.

**Fig. 2.**
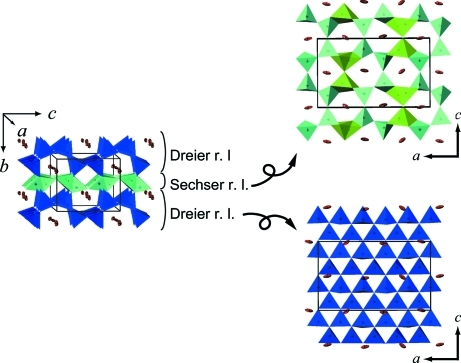
Drawings of the structure featuring alternate stacking of the dreier and sechser ring layers. The Si1—Si10 tetrahedra in the dreier ring layer are colored in dark blue, while the edge-sharing Si(Al)11-14 and Si(Al)l15-18 tetrahedra in the sechser ring layer are colored in pale blue and green, respectively. Sr (red) are located in the larger channels formed in the Si—Al—O—N skeleton.

**Fig. 3.**
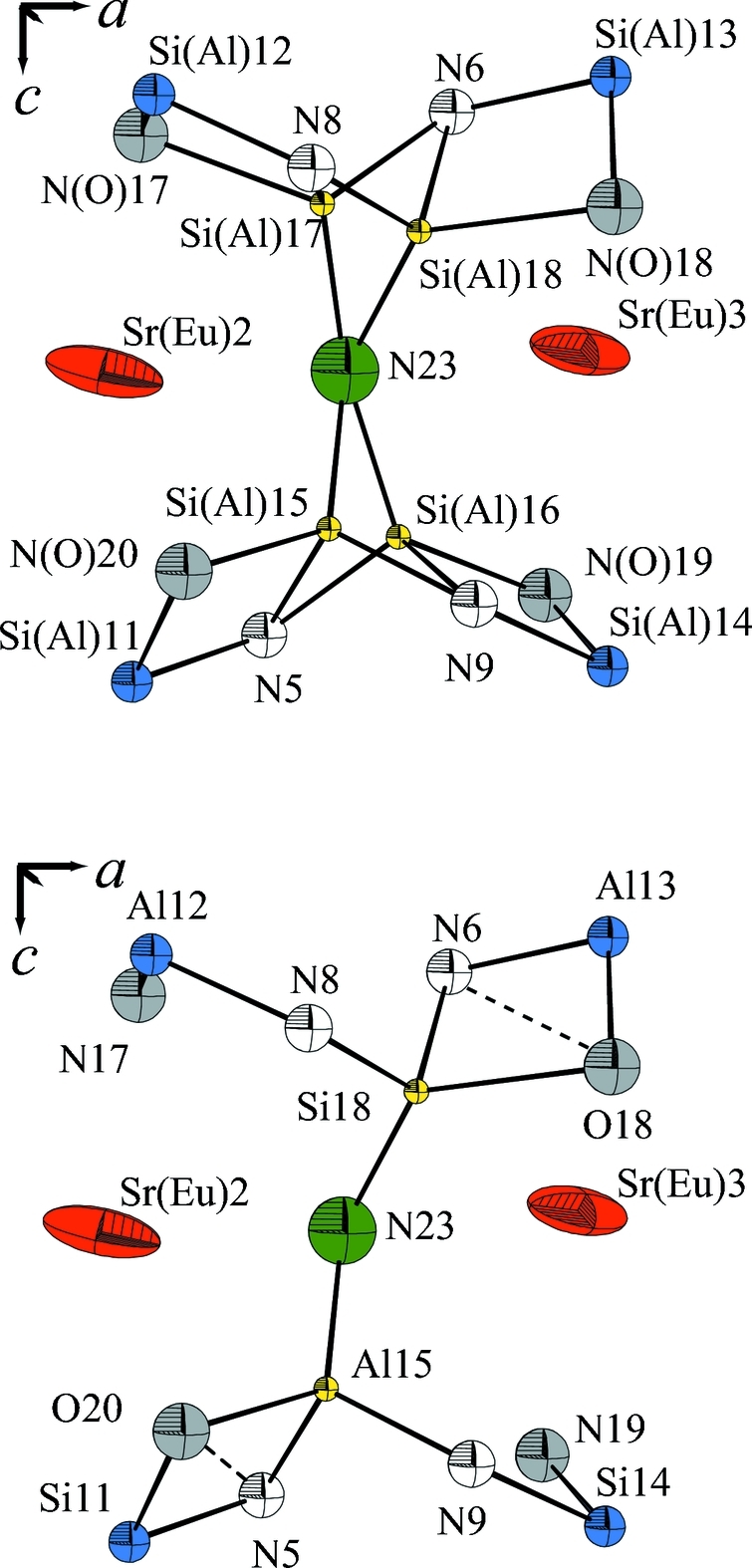
Local structure around the Si(Al)15-16 and Si(Al)17-18 split pairs in the sechser ring layer (top), and one of the possible conformations (bottom). The shared edge of tetrahedra is shown in dashed line in the bottom figure.

**Fig. 4.**
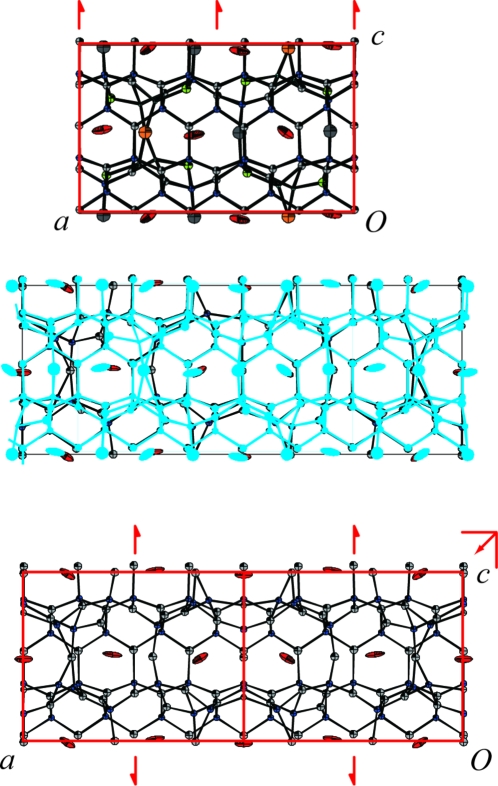
Comparison between the '3331' P1121 (top) and '5551' Pmn21 (bottom) structures projected along the b axis. Superposition of 3331 (blue) onto 5551 is shown in the middle where the screw axis at x=0 in 3331 overlaps with that at x=0.25 in 5551.

## supplementary crystallographic information

### Comment

The connectivity diagram of the asymmetric unit of structure is shown in Fig. 1. The Si and Al atoms are in tetrahedral coordination with N and O atoms, forming 'dreier' and 'sechser' ring layers, as shown in Fig. 2. These names follow the paper of Oeckler *et al.* (2009) on the Sr_5_Al_5+x_Si_21-x_N_35-x_O_2+x_:Eu^2+^ (x~0) structure. The Si(Al) tetrahedra are corner-shared in the dreier ring layer whereas an edge of each tetrahedron is shared with the other in the sechser ring layer. The alternating stacking of the dreier and sechser ring layers perpendicular to the *b* axis provides two channels with different sizes, running straight along the *a* axis. The crystals were usually grown in platelets developing {010} crystal faces, suggesting a slower growth rate along the stacking direction of the dreier and sechser ring layers.

The Si(Al)1-10 sites and N(O)1-16 sites in the dreier ring layer are almost exclusively occupied by Si and N, respectively, within the experimental error. The mean bond distance of the Si tetrahedra in the dreier ring layer is 1.76 (2) Å which is close to 1.72 Å as expected from the sum of ionic radii of Si^4+^ adn N^3-^ (Shannon, 1976). The N(O)17-20 sites are located at the boundary of the dreier and sechser ring layers and occupied equally by N and O atoms. The Si(Al)11-14 sites in the sechser ring layers contain Si and Al atoms. The Si(Al)15 -16 and Si(Al)17 -18 split pair sites in the sechser ring layers are half filled with Si or Al. The N(O)21-23 sites are located in the sechser ring layer and occupied by N almost exclusively within the experimental error. All the Si(Al) tetrahedra in sechser ring layer have at least one long bond of circa 1.80 Å, suggesting a statistical presence of Al—N bonds of ca. 1.85 Å (Shannon, 1976). This geometrical feature supports the result of population analysis for the structure containing two different atoms with similar scattering powers for X-rays at the same site.

The geometry around the Si(Al)15-16 and Si(Al)17-18 split pairs in the sechser ring layer is shown in Fig. 3. The configuration varies depending upon the real location of Si(Al) atom at either site of the split pair. The N23 site bridges two Si(Al) atoms in tetrahedral coordination.

The Sr atoms reside in the larger channel coordinating to 7 N(O) atoms at distances less than 3.1 Å. The Eu atoms displace 4-6% Sr atoms at Sr(Eu)2-3 sites, whereas none at Sr(Eu)1 within the experimental error. Since Sr(Eu)2-3 sites enriches O around themselves, a possible coupling of the Eu and O distributions in the structure can be pointed out.

Oeckler *et al.* (2009) reported that the composition of their Sr-bearing sialon Sr_5_Al_5+x_Si_21-x_N_35-x_O_2+x_:Eu^2+^ (x~0) can be expressed as '5551' , i.e., 5SrSiN_2_+5AlN+5Si_3_N_4_+1SiO_2_, when x=0. In this context, the composition of the present crystal, Sr_3_(Al_3+x_Si_13-x_) (N_21-x_O_2+x_) :Eu^2+ ^(x~0), corresponds to '6662'(='3331'), i. e., 6SrSiN_2_+6Si_3_N_4_+6AlN+2SiO_2_. The Eu analogue, Eu_3_(Al_3+x_Si_13-x_) (N_21-x_O_2+x_) (x=1/3) (Michiue *et al.*, 2009), is also close to 3331 where SrSiN_2_ should read EuSiN_2_.

The 5551 Sr-sialon has a commensurately modulated structure approximated by the orthorhombic *Pmn*2_1_ symmetry (Oeckler *et al., *2009). The unit cells of 5551 and 3331 Sr-sialons have similar *b* and *c* dimensions (~ 7.47 and ~9.04 Å, respectively), while they differ in *a* (~ 23.614 and ~14.756 Å, respectively). The two structures are considered as 8 and 5 times superstructure regarding the underlying parent structure with *a*_o_ =2.954 Å (Oeckler *et al. *2009), respectively. Actually, the ratio in *a*-length is 23.614/14.756 ~1.600 (=8/5), and the ratio in cell volume is 1602/995 ~1.610 (~ 8/5). If we apply this 8/5 rule to the composition, the 3331 compound can be expressed as Sr_4.8_(Al,Si)_25.6_(N,*O*)_36.8_ with respect to Sr_5_(Al,Si)_26_(N,*O*)_37_ of 5551. The ca. 4 at% relative deficiency of Sr in 3331 expressed as such is conspicuous compared with those of the other constituent atoms, providing an insight into a possible modulation mechanism in these structures.

The relative Sr-deficiency is a measure of Sr concentration per unit volume. Since the *b* and *c *lengths are approximately the same in 3331 and 5551, the Sr concentration per volume is reduced to that per length along the channel in which the Sr atoms reside. The mean Sr—Sr distances in 3331 and 5551 are 4.919 Å (=a_3331_/3=5a_o_/3) and 4.723 Å (=a_5551_/5=8a_o_/5) along the channel, respectively. The superimposed figure, as shown in Fig. 4, indicates that the Sr atoms faintly slip out of position as they depart further from a reference point on one of the two-fold screw axes. In another point of view, compounds 3331 and 5551 have 24 and 25 Sr atoms, respectively, in a single channel spanning 40a_o_. This one Sr-atom difference in number for every ~118 Å span along the Sr channel is an essential point that characterizes the structural difference between 3331 and 5551.

The 3331 and 5551 Sr-sialons have a structural similarity in the skeleton surrounding the Sr channels. The ADP ellipsoids of Sr in 3331 and 5551 compounds show a very large component along *a*, with the ratio of the largest/ smallest principal ADP components being close to 10, as shown in Figs. 1, 3 and 4. This extraordinary large anisotropy may suggest a possible occurrence of displacive modulation of the slippery Sr atoms along the channel. In the 5551 compound, this modulation has been evidenced in the diffuse scattering of commensurately modulated satellites along *a* (Oeckler *et al.*, 2009). On the other hand, many small crystal fragments picked up from the 3331 batch in the present study did not show any such diffuse nature. This could partly owe to the higher firing temperature of 2173 K to which the present 3331 batch was subjected.

The present analysis on the 3331 Sr-sialon was carried out independently and in a different approach against the study on the 3331 Eu-sialon by Michiue *et al.*, (2009). Although these 3331 sialons were finally found to have similar structures, a complicated microstructure like twins in crystals of Sr-sialon should be noted, as given in Refinement. This may suggest a presence of microstress in channels, which can be introduced by the replacement of Eu with Sr having a slightly larger ionic radius.

### Experimental

The powder sample with a starting composition of Sr_6_Si_27_Al_6_O_6_N_42_ was prepared from α-Si_3_N_4_ (SN—E10, Ube Industries Ltd.), SrO (Kojyundo Chemical Laboratory Co., Ltd.), and AlN (Type F, Tokuyama Co., Ltd.). The mixture was ground in the Si_3_N_4_ mortar and pestle. The powder sample was filled in h-BN crucible and fired in a graphite resistance furnace at 2173 K for 24 hours under 1 MPa nitrogen atmosphere. The chemical composition was not analyzed on this batch, but done on another Eu-doped (3.0 at%) batch using EPMA, resulting in Sr_5.82_Eu_0.18_Al_6.67_Si_26.77_N_41.50_O_4.11_. This composition can be rewritten as (Sr,Eu)_3_(Al_3+x_Si_13-x_)(N_21-x_O_2+x_) (x~0.06±0.32). A small amount of Eu was also detected from the X-ray diffraction analysis for the crystals sampled from the batch in which no Eu was included as starting components. This probably came from a contamination from the furnace or the crucible.

### Refinement

The reciprocal sections synthesized from the frame data indicated no significant diffuse scattering for any reflections. Several very weak reflections were found not coincident at the lattice points expected for the present structure. They were neglected because their locations differed from sample to sample. No significant departure from the metrical orthrhombicity was detected from the laboratory source and the synchrotron X-ray experiments. The Laue symmetry was well approximated by *mmm*. However, all trials of structure solution assuming the orthorhombic space groups were unsuccessful or only revealed that almost all the atom sites except for Sr should assume the split atom model. The best *R*1 factor was 0.11 for *P*2_1_22_1_.

The possibility of orthorhombic-mimicking monoclinic structure was then examined. From the systematic absence of reflections, possible space groups were limited to the noncentrosymmetric *P*112_1_ and centrosymmetric *P*112_1_/*m*. The latter was discarded in the course of solving structures. The refinement of the *P*112_1_ model assuming a single component in the crystal gave *R*1=0.0635 for 4384 reflections. A possible existence of twin components was suspected from a high *K* factor, defined as Mean[*F_o_*^2^]/Mean[*F_c_*^2^], of 2.6 in the region *F*_c_/*F*_c_(max) <0.033.

The point group of the crystal is 112 which is a merohedry in monoclinic crystal system. The symmetry element for the inversion twin is *m*' of the monoclinic holohedry, 112'/*m*'. In addition, since the crystal lattice is metrically orthorhombic, 'double twinning' can take place in which 'twin by merohedry' and 'twin by metric merohedry' occur at the same time (Coch, 2004; Nespolo, 2004). In the twin by metric merohedry, the twin element can be chosen from the symmetry elements of the orthorhombic holohedry, 2"/*m*"2"/*m*"2"/*m*". Possible combinations are categorized into five groups, (1) 112 (no twin), (2) 112/*m*' (inversion twin by merohedry), (3) *m*"12, 1*m*"2, and *m*"*m*"2 (reflection twin by metric merohedry), (4) 2"12, 12"2, and 2"2"2 (rotation twin by metric merohedry), and (5) *m*"2"2, 2"/*m*"12, 2"/*m*"2"/*m*"2, 2"12/*m*', 1*m*"2/*m*', and 2"/*m*"2"/*m*"2/*m*' (twin by double merohedry), each of which contains the same set of equivalent points. All these groups were examined through the least-squares procedure. The *R*1 factors are 0.0635 for group 1, 0.595 for group 2, 0.0481 for group 3, 0.475 for group 4, and 0.0447 for group 5. The double merohedry model (group 5) was finally adopted from comparing *R*1 factors, residual electrons, and *K* factors. The K factor improved significantly to 0.98 which is close to the ideal value of 1. The refined volume fractions of the individual twin components assuming 2"12/*m*' in the crystal are 28 (1)%, 22 (1)%, 21 (1)%, and 29 (1)%, where the second individual is related to the first by the two–fold rotation about *a*, and the third and forth are the inversions of the first and second, respectively.

Anisotropic ADPs for Sr(Eu) and isotropic ones for the other atoms were employed in the refinement. It was difficult to refine ADPs of all crystallographically independent Al(Si) and N(O) atom sites simultaneously, mainly due to insufficient high-angle diffraction data. As mentioned in Comment, the structure is composed of the dreier and sechser ring layers. The dreier ring layer is mainly composed of Si and N, while the sechser ring layers enriches Al and O at Si(Al) and N(O) sites, respectively, presenting more ionic bonding nature. It is also noted that the sechser ring layer contains Si(Al)15-16 and Si(Al)17-18 split atom pair sites with 50% population. Therefore the Al and Si atoms were classified into three groups each of which has similar circumstance in view of the structural-chemistry: (a) Si(Al)1 through Si(Al)10 in the dreier ring layer, having a tetrahedral coordination to N(O), (b) Si(Al)11 thorough Si(Al)14 in the sechser ring layer, and (c) Si(Al)15 through Si(Al)18 split pairs in the sechser ring layer. In a similar way, the N and O atoms are classified into (d) N(O)1 through N(O)16 with distorted planer 3-fold coordination in the dreier ring layer, (e) N(O)17 through N(O)20 with distorted planer 3-fold coordination in the sechser ring layer, and (f) N(O)21 through N(O)23 with distorted linear coordination with Si(Al) in the sechser ring layer and additional coordination to two Sr(Eu) atoms in the channel. The same ADP and the same population were assumed for Si(Al) atom sites belonging to the same group. Similar constraints were assumed for the N(O) atom sites. Preliminary population analysis suggested no Al in group (a), and no O in groups (d) and (f) within experimental errors. The other sites in groups (b), (c) and (e) were supposed to be almost equally occupied by the two atoms. To reduce ambiguities, fixed population numbers were assumed in the final stage of refinements except for Sr(Eu)2 and Sr(Eu)3. These constraints led the composition of the crystal to (Sr,Eu)_3_(Al_3+x_Si_13-x_)(N_21-x_O_2+x_) with x=0, which agreed with the EPMA analysis on a compound synthesized in similar conditions as mentioned in Experimental.

### Crystal data

**Table:**

Sr_3_Al_3_Si_13_N_21_O_2_	*F*(000) = 1001.0
*M**_r_* = 2081.94	*D*_x_ = 3.475 Mg m^−^^3^
Monoclinic, *P*112_1_	Mo *K*α radiation, λ = 0.71073 Å
Hall symbol: P 2c	Cell parameters from 3169 reflections
*a* = 14.7557 (5) Å	θ = 4.5–35.7°
*b* = 7.4627 (2) Å	µ = 9.05 mm^−^^1^
*c* = 9.0348 (3) Å	*T* = 296 K
β = 90°	Plate, light yellow
*V* = 994.89 (5) Å^3^	0.08 × 0.08 × 0.01 mm
*Z* = 1	

### Data collection

**Table:**

Bruker APEXII CCD diffractometer	4384 independent reflections
Radiation source: fine-focus sealed tube	3672 reflections with *I* > 2σ(*I*)
graphite	*R*_int_ = 0.036
φ and ω scans	θ_max_ = 27.5°, θ_min_ = 1.4°
Absorption correction: numerical (Meulenaer & Tompa, 1965)	*h* = −18→19
*T*_min_ = 0.532, *T*_max_ = 0.915	*k* = −9→9
11854 measured reflections	*l* = −11→11

### Refinement

**Table:**

Refinement on *F*^2^	Primary atom site location: structure-invariant direct methods
Least-squares matrix: full	Secondary atom site location: difference Fourier map
*R*[*F*^2^ > 2σ(*F*^2^)] = 0.045	*w* = 1/[σ^2^(*F*_o_^2^) + (0.0316*P*)^2^ + 7.9067*P*] where *P* = (*F*_o_^2^ + 2*F*_c_^2^)/3
*wR*(*F*^2^) = 0.105	(Δ/σ)_max_ < 0.001
*S* = 1.07	Δρ_max_ = 1.33 e Å^−^^3^
4384 reflections	Δρ_min_ = −1.25 e Å^−^^3^
162 parameters	Absolute structure: Flack (1983), 1942 Friedel pairs
1 restraint	Flack parameter: 0.00

### Special details

**Table:**

Geometry. Atom labels of Al and O are used for the Si(Al) and N(O) mixed sites, respectively, to distinguish them from the pure Si and N sites in Geometric parameters. All s.u.'s (except the s.u. in the dihedral angle between two l.s. planes) are estimated using the full covariance matrix. The cell s.u.'s are taken into account individually in the estimation of s.u.'s in distances, angles and torsion angles; correlations between s.u.'s in cell parameters are only used when they are defined by crystal symmetry. An approximate (isotropic) treatment of cell s.u.'s is used for estimating s.u.'s involving l.s. planes.
Refinement. Refinement of *F*^2^ against ALL reflections. The weighted *R*-factor wR and goodness of fit *S* are based on *F*^2^, conventional *R*-factors *R* are based on *F*, with *F* set to zero for negative *F*^2^. The threshold expression of *F*^2^ > 2σ(*F*^2^) is used only for calculating *R*-factors(gt) etc. and is not relevant to the choice of reflections for refinement. *R*-factors based on *F*^2^ are statistically about twice as large as those based on *F*, and *R*- factors based on ALL data will be even larger.

### Fractional atomic coordinates and isotropic or equivalent isotropic displacement parameters (Å^2^)

**Table:**

	*x*	*y*	*z*	*U*_iso_*/*U*_eq_	Occ. (<1)
Sr1	0.7496 (2)	0.7488 (2)	0.5178 (4)	0.0275 (3)	
Sr2	0.91735 (19)	1.2554 (2)	1.00147 (13)	0.0361 (8)	0.943 (11)
Eu2	0.91735 (19)	1.2554 (2)	1.00147 (13)	0.0361 (8)	0.057 (11)
Sr3	0.58236 (18)	1.2652 (2)	1.03237 (15)	0.0390 (8)	0.963 (11)
Eu3	0.58236 (18)	1.2652 (2)	1.03237 (15)	0.0390 (8)	0.037 (11)
Si1	0.9992 (3)	0.1230 (6)	0.6686 (5)	0.00785 (14)*	
Si2	0.8011 (3)	1.1316 (6)	0.6707 (4)	0.00785 (14)*	
Si3	0.5002 (3)	0.8635 (5)	0.8784 (5)	0.00785 (14)*	
Si4	0.6000 (3)	1.1307 (4)	0.6782 (4)	0.00785 (14)*	
Si5	0.6989 (3)	0.8634 (6)	0.8770 (4)	0.00785 (14)*	
Si6	0.8019 (3)	0.8726 (6)	0.1733 (5)	0.00785 (14)*	
Si7	0.6983 (3)	1.1443 (5)	0.3783 (4)	0.00785 (14)*	
Si8	0.6021 (3)	0.8640 (4)	1.1774 (3)	0.00785 (14)*	
Si9	0.8979 (3)	1.1420 (4)	0.3693 (4)	0.00785 (14)*	
Si10	0.8997 (3)	0.8467 (4)	0.8744 (3)	0.00785 (14)*	
Si11	0.9081 (4)	0.4859 (5)	0.6769 (4)	0.0089 (2)*	0.50
Al11	0.9081 (4)	0.4859 (5)	0.6769 (4)	0.0089 (2)*	0.50
Si12	0.9111 (3)	0.5575 (5)	0.3255 (4)	0.0089 (2)*	0.50
Al12	0.9111 (3)	0.5575 (5)	0.3255 (4)	0.0089 (2)*	0.50
Si13	0.5914 (3)	0.4893 (5)	0.3518 (4)	0.0089 (2)*	0.50
Al13	0.5914 (3)	0.4893 (5)	0.3518 (4)	0.0089 (2)*	0.50
Si14	0.5930 (4)	0.5559 (5)	0.7048 (4)	0.0089 (2)*	0.50
Al14	0.5930 (4)	0.5559 (5)	0.7048 (4)	0.0089 (2)*	0.50
Si15	0.7794 (5)	0.5102 (12)	0.8496 (9)	0.0031 (4)*	0.25
Al15	0.7794 (5)	0.5102 (12)	0.8496 (9)	0.0031 (4)*	0.25
Si16	0.7174 (5)	0.4400 (10)	0.8280 (8)	0.0031 (4)*	0.25
Al16	0.7174 (5)	0.4400 (10)	0.8280 (8)	0.0031 (4)*	0.25
Si17	0.7759 (5)	0.4455 (10)	0.1969 (8)	0.0031 (4)*	0.25
Al17	0.7759 (5)	0.4455 (10)	0.1969 (8)	0.0031 (4)*	0.25
Si18	0.7234 (5)	0.5127 (11)	0.1785 (9)	0.0031 (4)*	0.25
Al18	0.7234 (5)	0.5127 (11)	0.1785 (9)	0.0031 (4)*	0.25
N1	0.5017 (7)	0.9760 (14)	1.2397 (11)	0.0108 (3)*	
N2	0.6002 (7)	0.9021 (10)	0.9819 (9)	0.0108 (3)*	
N3	0.7020 (7)	0.9704 (14)	0.2475 (11)	0.0108 (3)*	
N4	1.0012 (10)	0.3442 (18)	0.7379 (15)	0.0108 (3)*	
N5	0.8092 (8)	1.3587 (16)	0.7131 (11)	0.0108 (3)*	
N6	0.6873 (9)	1.3573 (19)	0.3056 (15)	0.0108 (3)*	
N7	0.7989 (7)	1.1040 (13)	0.4784 (12)	0.0108 (3)*	
N8	0.8076 (9)	0.6538 (19)	0.2477 (15)	0.0108 (3)*	
N9	0.6904 (9)	0.6535 (18)	0.7795 (13)	0.0108 (3)*	
N10	0.9000 (8)	1.0199 (10)	0.7380 (8)	0.0108 (3)*	
N11	0.5017 (9)	0.3505 (18)	0.3017 (15)	0.0108 (3)*	
N12	0.9011 (8)	0.9851 (10)	0.2255 (8)	0.0108 (3)*	
N13	0.7984 (7)	0.8789 (13)	0.9806 (12)	0.0108 (3)*	
N14	0.5986 (8)	1.1229 (10)	0.4854 (8)	0.0108 (3)*	
N15	1.0006 (7)	0.8822 (13)	0.9770 (12)	0.0108 (3)*	
N16	0.7037 (7)	1.0287 (13)	0.7367 (11)	0.0108 (3)*	
N17	0.8937 (9)	0.3424 (17)	0.2743 (14)	0.0198 (8)*	0.50
O17	0.8937 (9)	0.3424 (17)	0.2743 (14)	0.0198 (8)*	0.50
N18	0.6009 (8)	0.6407 (13)	0.2163 (12)	0.0198 (8)*	0.50
O18	0.6009 (8)	0.6407 (13)	0.2163 (12)	0.0198 (8)*	0.50
N19	0.6054 (9)	0.3427 (16)	0.7602 (14)	0.0198 (8)*	0.50
O19	0.6054 (9)	0.3427 (16)	0.7602 (14)	0.0198 (8)*	0.50
N20	0.8832 (7)	0.6333 (16)	0.8160 (15)	0.0198 (8)*	0.50
O20	0.8832 (7)	0.6333 (16)	0.8160 (15)	0.0198 (8)*	0.50
N21	0.9172 (10)	0.5896 (13)	0.5118 (15)	0.0170 (7)*	
N22	0.5862 (11)	0.5887 (12)	0.5222 (15)	0.0170 (7)*	
N23	0.7617 (8)	0.4054 (11)	1.0138 (16)	0.0170 (7)*	

### Atomic displacement parameters (Å^2^)

**Table:**

	*U*^11^	*U*^22^	*U*^33^	*U*^12^	*U*^13^	*U*^23^
Sr1	0.0449 (6)	0.0107 (4)	0.0270 (6)	−0.0073 (12)	0.0216 (5)	−0.0071 (9)
Sr2	0.0732 (18)	0.0225 (7)	0.0126 (7)	−0.0157 (15)	0.0099 (9)	−0.0044 (5)
Eu2	0.0732 (18)	0.0225 (7)	0.0126 (7)	−0.0157 (15)	0.0099 (9)	−0.0044 (5)
Sr3	0.0664 (17)	0.0363 (11)	0.0143 (8)	0.0289 (14)	0.0104 (9)	−0.0003 (6)
Eu3	0.0664 (17)	0.0363 (11)	0.0143 (8)	0.0289 (14)	0.0104 (9)	−0.0003 (6)

### Geometric parameters (Å, °)

**Table:**

Sr1—N9	2.618 (13)	Si7—N7	1.764 (11)
Sr1—N8	2.682 (13)	Si7—N14	1.769 (11)
Sr1—N22	2.690 (16)	Si8—O18^iv^	1.703 (10)
Sr1—N21	2.745 (14)	Si8—N3^iv^	1.789 (11)
Sr1—N7	2.773 (9)	Si8—N2	1.789 (8)
Sr1—N16	2.955 (10)	Si8—N1	1.792 (11)
Sr1—N3	3.032 (10)	Si9—O17^i^	1.725 (13)
Sr2—N23^i^	2.557 (11)	Si9—N12	1.749 (8)
Sr2—O17^ii^	2.573 (13)	Si9—N7	1.784 (11)
Sr2—N21^iii^	2.702 (14)	Si9—N15^xii^	1.795 (11)
Sr2—N4^i^	2.764 (14)	Si10—O20	1.696 (13)
Sr2—N12^iv^	2.868 (7)	Si10—N15	1.775 (11)
Sr2—N10	2.970 (8)	Si10—N10	1.786 (8)
Sr2—N15	3.052 (10)	Si10—N13	1.792 (11)
Sr3—O19^i^	2.549 (12)	Al11—N21	1.687 (13)
Sr3—N22^v^	2.718 (16)	Al11—O20	1.710 (14)
Sr3—N2	2.760 (7)	Al11—N5^viii^	1.771 (12)
Sr3—N11^ii^	2.783 (13)	Al11—N4	1.819 (14)
Sr3—N23^i^	2.851 (11)	Al12—N4^vi^	1.685 (15)
Sr3—N6^iv^	2.994 (13)	Al12—O17	1.690 (13)
Sr3—N1	3.096 (10)	Al12—N21	1.702 (14)
Si1—N15^vi^	1.732 (12)	Al12—N8	1.829 (14)
Si1—N12^vii^	1.755 (12)	Al13—O18	1.672 (11)
Si1—N4	1.765 (14)	Al13—N22	1.711 (13)
Si1—N10^viii^	1.769 (11)	Al13—N11	1.740 (14)
Si2—N16	1.734 (11)	Al13—N6^viii^	1.773 (14)
Si2—N5	1.742 (13)	Al14—N22	1.671 (14)
Si2—N7	1.750 (12)	Al14—O19	1.678 (12)
Si2—N10	1.787 (11)	Al14—N9	1.747 (14)
Si3—N1^ix^	1.734 (11)	Al14—N11^x^	1.791 (15)
Si3—N11^x^	1.741 (15)	Al15—N23	1.697 (15)
Si3—N14^v^	1.752 (11)	Al15—N5^viii^	1.730 (14)
Si3—N2	1.770 (11)	Al15—N9	1.807 (16)
Si4—N14	1.743 (8)	Al15—O20	1.811 (13)
Si4—O19^i^	1.749 (13)	Al16—N9	1.700 (15)
Si4—N1^ix^	1.788 (12)	Al16—N5^viii^	1.811 (14)
Si4—N16	1.789 (12)	Al16—N23	1.820 (15)
Si5—N13	1.745 (11)	Al16—O19	1.906 (15)
Si5—N2	1.762 (11)	Al17—N8	1.687 (16)
Si5—N16	1.770 (11)	Al17—N23^xi^	1.694 (16)
Si5—N9	1.802 (14)	Al17—N6^viii^	1.762 (15)
Si6—N13^xi^	1.742 (12)	Al17—O17	2.026 (15)
Si6—N12	1.752 (12)	Al18—N6^viii^	1.716 (16)
Si6—N8	1.768 (15)	Al18—N8	1.745 (16)
Si6—N3	1.777 (11)	Al18—N23^xi^	1.783 (15)
Si7—N6	1.728 (15)	Al18—O18	2.072 (14)
Si7—N3	1.756 (11)		
			
N15^vi^—Si1—N12^vii^	106.4 (5)	O20—Si10—N15	115.0 (5)
N15^vi^—Si1—N4	112.0 (6)	O20—Si10—N10	117.7 (6)
N12^vii^—Si1—N4	108.2 (6)	N15—Si10—N10	104.5 (5)
N15^vi^—Si1—N10^viii^	110.2 (5)	O20—Si10—N13	100.0 (6)
N12^vii^—Si1—N10^viii^	113.0 (4)	N15—Si10—N13	113.6 (4)
N4—Si1—N10^viii^	107.1 (6)	N10—Si10—N13	106.0 (5)
N16—Si2—N5	114.3 (6)	N21—Al11—O20	111.8 (6)
N16—Si2—N7	105.9 (5)	N21—Al11—N5^viii^	118.4 (6)
N5—Si2—N7	109.5 (5)	O20—Al11—N5^viii^	91.8 (6)
N16—Si2—N10	110.7 (5)	N21—Al11—N4	118.3 (7)
N5—Si2—N10	108.8 (5)	O20—Al11—N4	108.3 (6)
N7—Si2—N10	107.3 (5)	N5^viii^—Al11—N4	104.8 (6)
N1^ix^—Si3—N11^x^	110.2 (6)	N4^vi^—Al12—O17	113.7 (7)
N1^ix^—Si3—N14^v^	110.2 (5)	N4^vi^—Al12—N21	111.3 (7)
N11^x^—Si3—N14^v^	105.0 (6)	O17—Al12—N21	114.4 (6)
N1^ix^—Si3—N2	106.4 (5)	N4^vi^—Al12—N8	106.8 (6)
N11^x^—Si3—N2	112.0 (6)	O17—Al12—N8	98.1 (7)
N14^v^—Si3—N2	113.1 (4)	N21—Al12—N8	111.7 (6)
N14—Si4—O19^i^	117.0 (5)	O18—Al13—N22	111.7 (5)
N14—Si4—N1^ix^	106.6 (5)	O18—Al13—N11	106.0 (6)
O19^i^—Si4—N1^ix^	108.0 (6)	N22—Al13—N11	117.3 (7)
N14—Si4—N16	107.0 (5)	O18—Al13—N6^viii^	97.8 (7)
O19^i^—Si4—N16	102.8 (6)	N22—Al13—N6^viii^	119.2 (7)
N1^ix^—Si4—N16	115.9 (4)	N11—Al13—N6^viii^	102.4 (5)
N13—Si5—N2	113.3 (5)	N22—Al14—O19	116.1 (6)
N13—Si5—N16	107.7 (5)	N22—Al14—N9	111.7 (7)
N2—Si5—N16	107.7 (5)	O19—Al14—N9	101.0 (7)
N13—Si5—N9	112.2 (6)	N22—Al14—N11^x^	112.3 (7)
N2—Si5—N9	110.4 (6)	O19—Al14—N11^x^	108.0 (7)
N16—Si5—N9	105.0 (5)	N9—Al14—N11^x^	106.9 (6)
N13^xi^—Si6—N12	106.4 (5)	N23—Al15—N5^viii^	111.2 (7)
N13^xi^—Si6—N8	114.0 (6)	N23—Al15—N9	117.9 (7)
N12—Si6—N8	107.5 (6)	N5^viii^—Al15—N9	108.8 (7)
N13^xi^—Si6—N3	109.9 (5)	N23—Al15—O20	120.7 (7)
N12—Si6—N3	113.3 (5)	N5^viii^—Al15—O20	89.8 (7)
N8—Si6—N3	106.0 (6)	N9—Al15—O20	104.8 (7)
N6—Si7—N3	115.2 (6)	N9—Al16—N5^viii^	109.9 (7)
N6—Si7—N7	115.5 (6)	N9—Al16—N23	117.1 (6)
N3—Si7—N7	101.2 (5)	N5^viii^—Al16—N23	102.3 (6)
N6—Si7—N14	102.3 (5)	N9—Al16—O19	94.1 (6)
N3—Si7—N14	109.1 (4)	N5^viii^—Al16—O19	109.7 (6)
N7—Si7—N14	113.9 (5)	N23—Al16—O19	123.7 (6)
O18^iv^—Si8—N3^iv^	111.7 (6)	N8—Al17—N23^xi^	117.6 (7)
O18^iv^—Si8—N2	111.1 (5)	N8—Al17—N6^viii^	113.5 (8)
N3^iv^—Si8—N2	107.0 (5)	N23^xi^—Al17—N6^viii^	112.7 (7)
O18^iv^—Si8—N1	112.5 (6)	N8—Al17—O17	91.0 (7)
N3^iv^—Si8—N1	111.3 (4)	N23^xi^—Al17—O17	112.1 (7)
N2—Si8—N1	102.8 (5)	N6^viii^—Al17—O17	107.6 (7)
O17^i^—Si9—N12	102.2 (5)	N6^viii^—Al18—N8	112.9 (8)
O17^i^—Si9—N7	112.5 (6)	N6^viii^—Al18—N23^xi^	110.7 (7)
N12—Si9—N7	109.0 (5)	N8—Al18—N23^xi^	110.1 (7)
O17^i^—Si9—N15^xii^	112.8 (6)	N6^viii^—Al18—O18	86.0 (6)
N12—Si9—N15^xii^	108.2 (5)	N8—Al18—O18	106.5 (7)
N7—Si9—N15^xii^	111.6 (4)	N23^xi^—Al18—O18	128.4 (7)

Symmetry codes: (i) *x*, *y*+1, *z*; (ii) *x*, *y*+1, *z*+1; (iii) −*x*+2, −*y*+2, *z*+1/2; (iv) *x*, *y*, *z*+1; (v) −*x*+1, −*y*+2, *z*+1/2; (vi) −*x*+2, −*y*+1, *z*−1/2; (vii) −*x*+2, −*y*+1, *z*+1/2; (viii) *x*, *y*−1, *z*; (ix) −*x*+1, −*y*+2, *z*−1/2; (x) −*x*+1, −*y*+1, *z*+1/2; (xi) *x*, *y*, *z*−1; (xii) −*x*+2, −*y*+2, *z*−1/2.
